# High PD-L1 Expression on Tumor Cells Indicates Worse Overall Survival in Advanced Oral Squamous Cell Carcinomas of the Tongue and the Floor of the Mouth but Not in Other Oral Compartments

**DOI:** 10.3390/biomedicines9091132

**Published:** 2021-09-01

**Authors:** Łukasz Jan Adamski, Anna Starzyńska, Paulina Adamska, Michał Kunc, Monika Sakowicz-Burkiewicz, Giulia Marvaso, Daniela Alterio, Aleksandra Korwat, Barbara Alicja Jereczek-Fossa, Rafał Pęksa

**Affiliations:** 1 Department of Oral Surgery, Medical University of Gdańsk, 7 Dębinki Street, 80-211 Gdańsk, Poland; lukaszadamski@gumed.edu.pl (Ł.J.A.); paulina.adamska@gumed.edu.pl (P.A.); 2 Department of Pathology, Medical University of Gdańsk, 17 Smoluchowskiego Street, 80-214 Gdańsk, Poland; michal.kunc@gumed.edu.pl (M.K.); aleksandra.korwat@gumed.edu.pl (A.K.); rafal.peksa@gumed.edu.pl (R.P.); 3 Department of Molecular Medicine, Medical University of Gdańsk, 7 Dębinki Street, 80-211 Gdańsk, Poland; ssak@gumed.edu.pl; 4 Department of Oncology and Hemato-Oncology, University of Milan, 7 Festa del Perdono Street, 20-112 Milan, Italy; giulia.marvaso@ieo.it (G.M.); barbara.jereczek@ieo.it (B.A.J.-F.); 5 Division of Radiotherapy, IEO European Institute of Oncology, IRCCS, 435 Ripamonti Street, 20-141 Milan, Italy; daniela.alterio@ieo.it

**Keywords:** programmed cell death-ligand 1, PD-L1, interleukin 33, IL-33, oral squamous cell carcinoma, prognosis, immunohistochemistry

## Abstract

The markers of the tumor microenvironment (TME) are promising prognostic and predictive factors in oral squamous cell carcinoma (OSCC). The current study aims to analyze the immunohistochemical expression of programmed cell death-ligand 1 (PD-L1) and interleukin-33 (IL-33) in a cohort of 95 chemonaïve OSCCs. PD-L1 and IL-33 were assessed separately in tumor cells (TCs) and tumor-infiltrating lymphocytes (TILs). High PD-L1 expression in TILs was associated with better overall survival (OS) in univariate analysis. Tumors localized in the floor of the oral cavity and tongue tended to have a lower percentage of PD-L1-positive TCs when compared to other locations. PD-L1 expression on TCs had no prognostic significance when the whole cohort was analyzed. However, along with the T descriptor (TNM 8^th^), it was included in the multivariable model predicting death in carcinomas of the floor of the oral cavity and tongue (HR = 2.51, 95% CI = 1.97–5.28). In other locations, only nodal status was identified as an independent prognostic factor in multivariate analysis (HR = 0.24, 95% CI = 0.08–0.70). Expression of IL-33 had no impact on survival, but it was differently expressed in various locations. In conclusion, the prognostic significance of PD-L1 in oral cancer depends on the tumor site and type of cell expressing immune checkpoint receptor (TCs vs. TILs).

## 1. Introduction

Oral squamous cell carcinoma (OSCC) accounts for 95% of all malignancies developing in the oral cavity. It predominantly affects males over 50 years of age and has a causal relationship with tobacco smoking and alcohol consumption [1]. Despite the substantial progress which has been made in OSCC treatment, overall survival (OS) has not improved significantly in the last few decades. The treatment for OSCC is multimodal, with surgery usually being the treatment of choice. Its clinical course mainly depends on the stage of diagnosis, tumor location, and the feasibility of radical surgical resection. Carcinomas of the tongue and floor of the oral cavity are frequently locally advanced at the time of the first presentation and show a worse prognosis than other oral cancers. Nevertheless, other factors related to tumor biology and its immune microenvironment may also influence the prognosis or be the targets for personalized therapy [2,3,4].

The tumor microenvironment (TME) is a dynamic ecosystem consisting of cancer cell, stromal cells, blood vessels, extracellular matrix, and numerous types of immune cells which exhibit complex reciprocal interactions [5,6]. Cancer cells and immune cells may interact via programmed cell death ligand-1 (PD-L1/B7-H1/CD274) and its receptor programmed cell death-protein 1 (PD-1) [7]. PD-L1 expression by cancer cells is one of the mechanisms of immune response evasion, as it activates immune checkpoint protein PD-1 on cytotoxic CD8+ T lymphocytes and reduces their activity (a phenomenon which is called “exhaustion”) [8]. Perplexingly, PD-L1 may be also expressed by immune cells, but the exact role of this process is not fully understood. In some cancers, PD-L1 expression on immune cells is associated with favorable outcomes, whereas in others it is a poor prognostic factor [9,10]. The PD-L1/PD-1 axis is a target of multiple drugs (immune checkpoint inhibitors), which have substantially improved survival, for example, in advanced non-small-cell lung carcinoma and melanoma [11,12,13]. To date, several studies aimed to assess the prognostic significance of the PD-L1/PD-1 axis in OSCC [2]. Combination of anti-PD-1 immunotherapy (pembrolizumab or nivolumab) with chemotherapy and radiation therapy improves outcomes in OSCC [14]. Conflicting data exists on the prognostic significance of PD-1/PD-L1 expression in OSCC. Predictive significance is also unclear, but both PD-L1 expressors and non-expressors benefit from anti-PD-L1 therapy [13,15].

Interleukin-33 (IL-33), a member of the IL-1 family, is a chromatin-associated cytokine released from the nucleus into extracellular space triggered by stress or necrosis, and serves as an endogenous danger signal (alarmin) [16]. Its role is to activate the immune system in response to tissue damage via interactions with its receptor, ST2, expressed by various immune cells. IL-33 is constitutively expressed by a variety of cells, including endothelial cells, fibroblasts, and epithelial cells, but it may be up-regulated in reaction to stresses. IL-33 plays a role in chronic inflammatory diseases like asthma and Crohn’s disease, and bacterial, fungal, and parasitic infections [16,17,18]. Head and neck carcinomas overexpressing IL-33 in cancer cells and carcinoma-associated fibroblasts (CAFs) were characterized by higher invasiveness and worse outcomes [19]. Moreover, the IL-33/ST2 axis influences other elements of the TME, since it modifies the activity of T-helper lymphocytes, regulates the production of IL-4, IL-5, and IL-13, and angiogenesis [20,21]. Finally, IL-33 modulates PD-1/PD-L1 expression in the cancer microenvironment, and preliminary studies suggest that co-targeting of IL-33 and immune checkpoint receptors may improve the outcomes of immunotherapy [22]. The role of IL-33 in cancer is most likely context-dependent because it demonstrates either an anti-tumorigenesis effect (e.g., pancreatic cancer, ovarian, and colon cancer) or a pro-tumorigenesis effect (e.g., breast cancer, lung cancer) [20,23]. However, our knowledge about IL-33 expression in OSCC is scant [24,25].

In the current study, we aimed to evaluate PD-L1 and IL-33 expression in patients with OSCC in relation to clinical characteristics and survival.

## 2. Materials and Methods

### 2.1. Study Group

Medical records of 109 patients diagnosed with OSCC, treated at the University Clinical Center Medical University of Gdańsk, Poland, between 2007–2012 were analyzed. The inclusion criteria of this study included histopathologically confirmed OSCC and available treatment-naïve histopathological specimens (biopsy or resection). Patients without survival data were excluded (n = 14). Finally, ninety-five patients (n = 95) were included in the study. Basic demographic and clinicopathological data (age, gender, addictions, treatment methods, cancer localization, grading, staging, recurrence, death, follow-up, and survival rate) were collected. The staging in all cases was determined within one month after the first presentation, and adjusted according to the American Joint Committee on Cancer (AJCC) 8th edition of the TNM classification for the sake of the current study. Patients’ data were fully anonymized. The local Bioethical Committee of the Medical University of Gdańsk approved the protocol of the study (approval No NKBBN/59/2016).

### 2.2. Specimen Preparation and Immunohistochemistry

Formalin-fixed paraffin-embedded (FFPE) tissue blocks were collected from tumor resection or, in the case of patients treated with neoadjuvant radiation or chemotherapy, from treatment-naïve biopsy (if applied) after the first presentation. Tissue microarrays (TMA) were prepared with a Manual Tissue Arrayer MTA 1 (Beecher Instruments Inc., Sun Prairie, WI, USA). Two representative cores, both of 0.4 cm diameter, were obtained from each case.

IHC was performed using the Ventana G11 system (CONFIRM™, Ventana Medical Systems, Tucson, AZ, USA). TMAs were stained with anti-PD-L1 antibody (rabbit monoclonal antibody, E1L3N, Cell Signaling, Danvers, MA, USA) and anti-IL-33 antibody (rabbit monoclonal antibody, MAB36252; Clone 1061A; R&D Systems, Inc., Bio-Techne, MN, USA).

**Figure 1:**
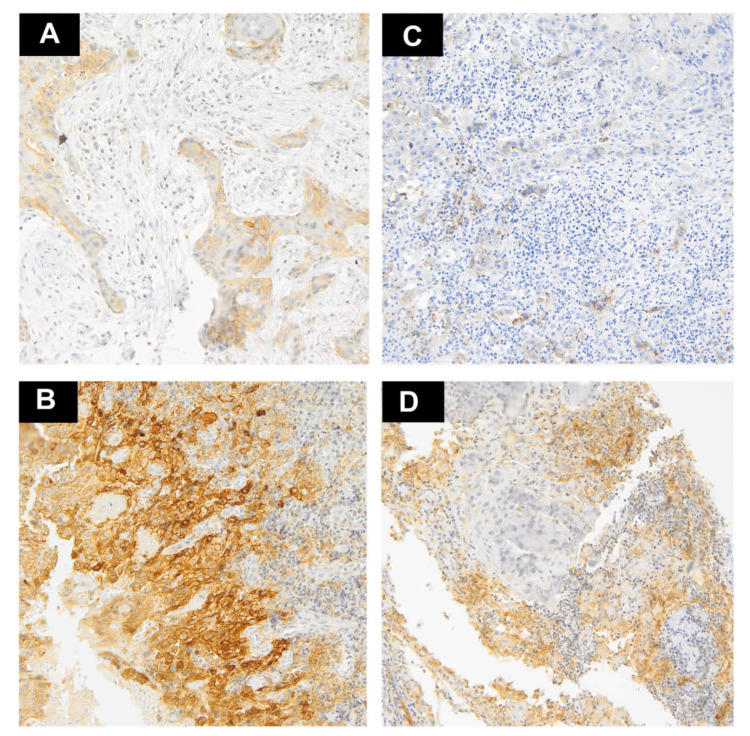
Representative examples of PD-L1 staining (magnification ×10). (**A**) A few tumor cells with weak expression of PD-L1; (**B**) Intense expression of PD-L1 in the majority tumor cells and weak staining in TILs at the tumor–stroma interface; (**C**) Very weak expression of PD-L1 on single lymphocytes and negative on cancer cells; (**D**) High expression of PD-L1 on TILs at the tumor–stroma interface and negative on tumor cells.

**Figure 2:**
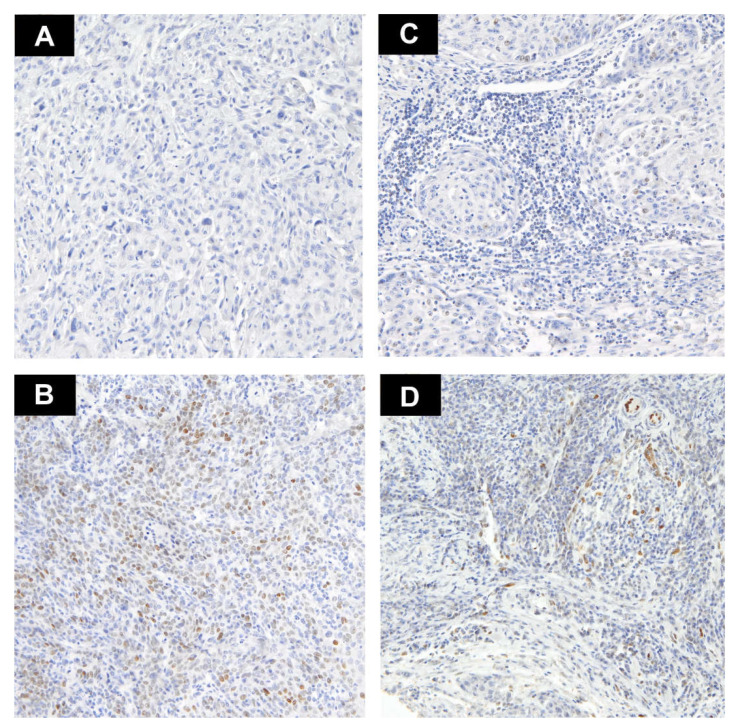
Representative examples of IL-33 staining (magnification ×10). (**A**) Tumor cells lacking IL-33 expression (no staining); (**B**) Positive nuclear staining with heterogeneous intensity in tumor cells; (**C**) TILs with negative IL-33 staining; (**D**) Weak positive nuclear staining in TILs and negative in TCs.

The proportion of positive cells was established by calculating the number of stained tumor cells (TCs) and tumor-infiltrating lymphocytes (TILs) divided by the total number of each type of cells. Two pathologists (AK and RP) experienced in PD-L1 expression evaluation assessed the stainings. When the interpretations differed, the pathologists made decisions by consensus. Only membranous PD-L1 expression was considered positive in TCs, whereas cytoplasmic and/or membranous reaction were considered positive in TILs. Only nuclear IL-33 expression was considered positive. Histologically normal tonsil was used for the positive control. For each patient, the results from two cores were used. The percentage of positively staining cells was estimated in each core and an average score was utilized in the further analyzes. The cut-off for high PD-L1 expression was established depending on the median of the percentage of positively staining cells as >10% in TCs and >20% in TILs (Figure 1A–D). IL33 was divided into two groups—no expression and positive expression (Figure 2A–D). Subsequently, we compared the agreement between two cores in the binary classification of PD-L1 and IL-33 by Cohen’s kappa coefficient to assess the heterogeneity of markers expression.

### 2.3. Statistical Analysis

Statistical analysis was performed using the STATISTICA 13.3 (TIBCO, Palo Alto, CA, USA; licensed to the Medical University of Gdańsk) and R statistical environment [26]. Kaplan–Meier curves were plotted using the “survminer” and “ggsci” packages [27,28]. The associations between analyzed markers and clinicopathological characteristics were assessed by the Mann–Whitney U test for continuous variables. Categorical variables were compared by the chi-square test and Fisher’s exact test when applicable. Cohen’s kappa coefficient was calculated to assess the reproducibility across the two cores incorporated in the TMA. Kaplan–Meier curves were plotted to assess overall survival (OS) and compared by log-rank test. Hazard ratios were estimated with the Cox proportional hazard regression. The backward selection was employed to create a multivariable model predicting death and to eliminate non-significant variables at *p* ≤ 0.05. All tests were considered statistically significant as *p* ≤ 0.05.

## 3. Results

### 3.1. PD-L1 Expression

Forty-four (46.31%) cases demonstrated positive expression of PD-L1 in > 10% of TCs. The mean percentage of positive cells was 21.88%, median 10%. Tumors localized in the floor of the oral cavity and tongue tended to have a lower percentage of PD-L1-positive TCs when compared to other locations (*p* = 0.019, Mann–Whitney U). There was a trend toward lower PD-L1 expression and the presence of nodal metastases (*p* = 0.015, Mann–Whitney U). Analogous findings were noted if PD-L1 was assessed as a binary variable (low/high expression). There was no association with gender, T stage, grade, history of smoking, or alcohol abuse. The summary of clinicopathological features with relation to analyzed biomarkers is presented in Table 1.

Thirty-one (31.63%) cases displayed PD-L1 positivity in >20% of TILs (high expression). High PD-L1 expression on TILs was associated with the absence of lymph node metastases and lower stage. No association was found between the expression of PD-L1 in TILs and other analyzed clinicopathological variables.

The agreement between cores was moderate in terms of PD-L1 expression in TCs (Cohen’s kappa = 0.645, 95% CI = 0.492–0.799), and fair in terms of PD-L1 expression in TILs (Cohen’s kappa = 0.335, 95% CI = 0.126–0.545).

### 3.2. IL-33 Expression

Fifteen cases (15.79%) demonstrated IL-33 expression in >1% of TCs nuclei (mean 1.08%, median 0%, max. 30%). Cancers of the tongue and the floor of the oral activity expressed IL-33 less commonly (*p* = 0.001, chi-square). Stage 3–4 OSCCs tended to express IL-33 more commonly than lower stage tumors, but this finding had borderline statistical significance (*p* = 0.057, chi-square). No other clinicopathological variables showed association with IL-33 expressed by TCs.

Positive expression of IL-33 in TILs was observed in 18 cases (18.94%). Mean percentage was 0.5% (median 0%, maximum 7%). Expression of IL-33 in TILs was less common in cancers of the tongue and the floor of the oral cavity (*p* = 0.055, chi-square), but no other association between IL-33 and clinicopathological variables was found (Supplementary Table S1).

Expression repeatability between IL-33 cores was substantial in terms of TCs (Cohen’s kappa = 0.707, 95% CI = 0.487–0.927), and moderate in the case of TILs (Cohen’s kappa = 0.493, 95% CI 0.235–0.752). No correlation was found between PD-L1 and IL-33 expression.

### 3.3. Survival Analysis

#### 3.3.1. Whole Cohort

**Figure 3:**
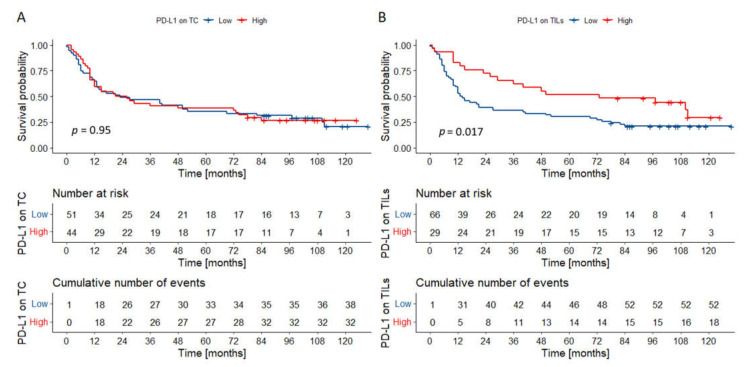
Kaplan–Meier curves for overall survival according to PD-L1 expression on TCs (**A**), and TILs (**B**). PD-L1 expression on TCs had no impact on the outcomes in the whole cohort. High PD-L1 expression on TILs was associated with superior overall survival. *p* values were calculated with the log-rank test. Abbreviations: TC—tumor cells; TILs—tumor-infiltrating lymphocytes.

The mean follow-up was 3.83 years, the minimum was 24 days, and the maximum was 10.87 years. The 5-year OS was 36.65%. The univariate Cox’s proportional hazard analysis (Table 2) demonstrated the association between survival and grade, stage, nodal metastases, and PD-L1 expression on TILs. Expression of IL-33 and PD-L1 in TCs had no impact on survival (Figure 3A for PD-L1, Supplementary Figures S1–S3 for IL-33). High PD-L1 expression in TILs was associated with better OS (HR = 0.475, 95% CI = 0.281–0.805; Figure 3B), but it was not retained in the multivariate Cox regression model predicting outcomes. Only the presence of nodal metastases was incorporated in the multivariable model predicting death.

#### 3.3.2. Prognostic Significance of PD-L1 and IL-33 Expression in Various Locations

Due to the significant differences in PD-L1 expression in cancers of the tongue and floor of the oral cavity, we decided to create separate multivariable Cox’s regression models that predict outcomes and take into consideration cancer location.

**Figure 4:**
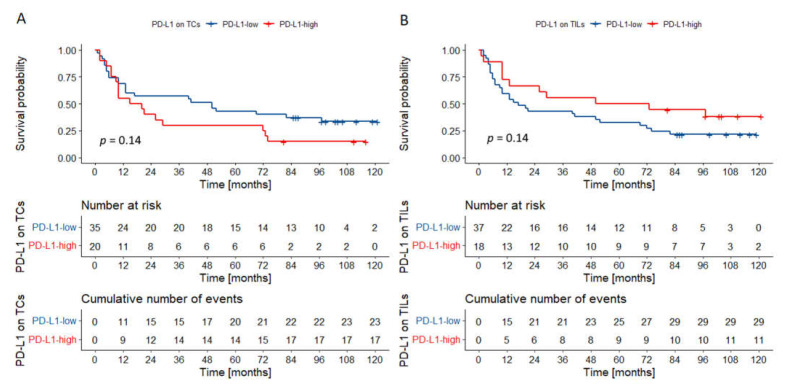
Overall survival probability curve according to PD-L1 expression on TCs (**A**) and TILs (**B**) in OSCCs of the tongue/floor of the oral cavity (**A**,**B**). There was a trend towards worse overall survival in cancers of the tongue/floor of the oral cavity with high PD-L1 expression on TCs. An opposite trend was observed for PD-L1 expression on TILs. *p* values were calculated with the log-rank test. Abbreviations: TCs—tumor cells; TILs—tumor infiltrating lymphocytes; OSCC—oral squamous cell carcinoma.

**Figure 5:**
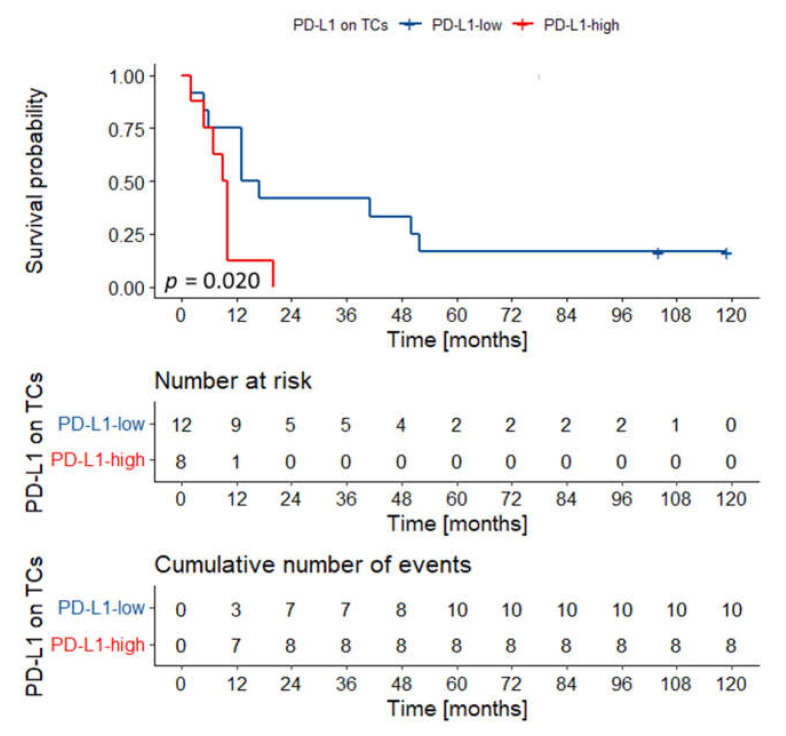
Overall survival probability curve according to PD-L1 expression on TCs in T3–T4 tumors of the tongue/floor of the oral cavity. Tumors with high PD-L1 expression on TCs had dismal outcomes. *p* value was calculated with the log-rank test. Abbreviations: TCs—tumor cells.

In the group of cancers of the tongue and the floor of the oral cavity, two variables were incorporated in the final model: T category and PD-L1 expression on TCs (Table 3). Interestingly, in univariate analysis, PD-L1 expression on TCs and TILs had a statistically borderline impact on survival (Figure 4). Especially poor outcomes were observed in the group of T3–4 tumors highly expressing PD-L1 on TCs (Figure 5).

**Figure 6:**
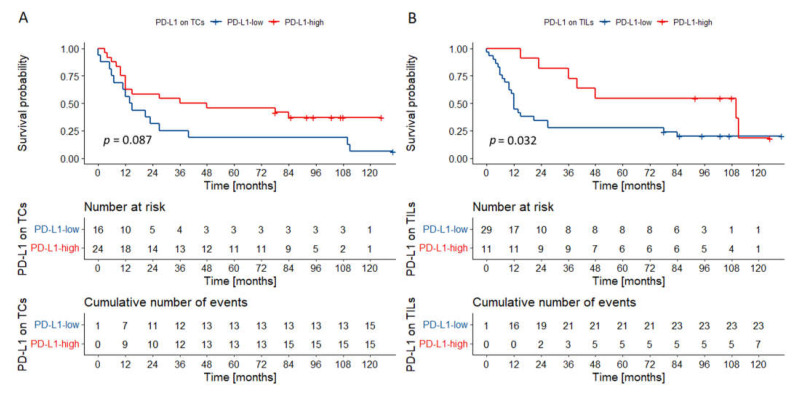
Overall survival probability curves according to PD-L1 expression on TCs (**A**) and TILs (**B**) in other oral compartments. There was a trend toward better survival in tumors characterized by high PD-L1 expression on TCs. Tumors rich in PD-L1-positive TILs had superior outcomes. *p* values were calculated with the log-rank test. Abbreviations: TCs—tumor cells; TILs—tumor infiltrating lymphocytes.

In cancers located in other parts of the oral cavity, only the presence of nodal metastases was incorporated in the multivariable model. Importantly, in univariate analysis, PD-L1 expression on TILs was associated with better outcomes (Figure 6).

## 4. Discussion

The oral cavity is in constant contact with the external environment. There are numerous reactions here that are designed to protect the body against harmful factors. The importance of the immune tumor microenvironment and tumor immunology in the prognosis of patients with OSCCs and other head and neck malignancies is becoming increasingly recognized [2,3,29,30]. This is the first study to co-analyze PD-L1 and IL-33 protein expression on TCs and TILs in OSCC. We demonstrated that the level of PD-L1 expression on TCs varies depending on its location—cancers of the tongue and the floor of the oral cavity show lower expression than cancers of other parts. The expression of PD-L1 on TCs was not related to OS in the entire cohort. However, the expression of PD-L1 on TCs appears to have opposite effects in cancers of different locations, which “canceled out” when analyzing the entire cohort. Higher PD-L1 expression on TCs in the carcinomas of the tongue/floor of the oral cavity was associated with a worse OS, especially in cancers of higher T category in TNM. In other locations, higher PD-L1 expression on TCs was associated with a better prognosis (a trend, but not statistically significant). On the other hand, higher PD-L1 expression on TILs was associated with a lower frequency of nodal metastases. Moreover, it was associated with longer OS in the univariate analysis, but this effect was not maintained in the multivariate analysis. Most likely, this is due to the very strong correlation of PD-L1 on TILs and nodal metastases. The latter was identified as the single most important prognostic factor in the whole cohort. These findings are supported by studies on large populations, which demonstrated that the number of metastatic lymph nodes and its characteristics (e.g., the presence of extranodal extension) are critical predictors of survival in OSCC [31,32].

In other works concerning OSCC, PD-L1 expression on TCs was observed with variable frequency ranging from approximately 10 to 90% of cases [33,34,35,36,37,38,39,40,41,42,43,44,45,46,47,48,49,50,51,52,53,54,55,56,57,58,59,60,61,62,63,64,65,66]. The studies differed in the size of the study group, selection of patients, antibody clones used, and the way of assessing PD-L1 expression; the two latter factors in particular significantly influence the final results of the study. In the Supplementary Table S2, we briefly present the previous studies analyzing PD-L1 expression in OSCC with the emphasis on methodology and prognostic effects.

Most studies to date, similarly to ours, are based on the heterogeneous cohorts of tumors located throughout the oral cavity, with a few exceptions focused on certain oral compartments, e.g., squamous cell carcinoma of the tongue. Multiple studies assessing survival in OSCCs demonstrated that high PD-L1 expression on TCs is associated with worse outcomes. Strati et al. [67] demonstrated that PD-L1 overexpression on circulating TCs was associated with inferior progression-free survival and OS in head and neck cancers. Nevertheless, a few studies showed contrary results or no association between PD-L1 expression and survival. The results of our study suggest that these discrepancies may originate from skewed distribution of cancer location in analyzed cohorts, as PD-L1 expression on TCs seems to have different prognostic effects in various compartments of the oral cavity. The site-dependent differences in the TME composition of head and neck carcinomas were previously reported by Green et al. [68], who observed a higher prevalence of TILs in oropharyngeal cancers compared to other locations. Interestingly, PD-L1 is physiologically expressed on the masticatory mucosa of the oral cavity, whereas other epithelia do not constitutively express PD-L1 [69]. Additionally, oral compartments represent ecological niches inhabited by a variety of microbiota modulating the local immune microenvironment [70,71]. For instance, *Porphyromonas gingivalis* induces the expression of PD-L1 in OSCC cells in vitro [53]. Finally, the immune landscape of HPV- and carcinogen-driven head and neck carcinomas differ in some aspects [72]. All these factors may contribute to distinct immune characteristics of OSCCs of various sites.

However, the prognostic significance of PD-L1 expression in TCs may also depend on other factors, especially the TME context in certain parts of the oral cavity [73]. Takahashi et al. [42] demonstrated that patients with high PD-L1 expression and abundant CD4+ T-cells have better outcomes than those with low CD4+ T-cell infiltration. Other research showed that high infiltration by CD4+ and CD8+ and high CD8+/FOXP3+ ratio lymphocytes were associated with positive expression of PD-L1 on TCs [33]. Other factors which influence PD-L1 expression in OSCC include gender, since some studies showed more PD-L1-negative tumors in males than in females [37,39]. Hanna et al. [44] reported that PD-L1 expression was associated with better outcomes in young females with OSCC. In another study, PD-L1 expression on TCs was more common in non-smokers and non-drinkers [52].

Even more perplexing is the role of PD-L1 expression on immune cells in OSCC. Previous translational research demonstrated that PD-L1-positive macrophages induce anergy in CD4+ and CD8+ T-cells in OSCC TME [56,57]. However, the current study demonstrates the more favorable prognosis of OSCC infiltrated by the high number of PD-L1-positive TILs. This effect was strongly correlated with the absence of nodal metastases. These results are consistent with the study by Kim et al. [10] which analyzed a large cohort of head and neck squamous cell carcinomas (including 204/402 oral cancers), and reported that PD-L1 expression on TILs, but not on TCs, was a favorable prognostic factor. Similar associations were noted in laryngeal cancer [74]. Better outcomes and lack of nodal metastases in tumors rich in PD-L1 positive TILs in OSCCs may be related to preexistent anti-tumor adaptive immune response [75].

In our study, IL-33 was rarely expressed in OSCC and had no significant impact on patients’ survival. In the only study so far analyzing IL-33 in oral cancer, Ishikawa et al. [24] evaluated IL-33 expression in squamous cell carcinoma of the tongue [24]. The authors showed that high IL-33 expression in tumor cells was associated with local and nodal recurrence. They used a different antibody clone (IL-33, MBS150331, rabbit polyclonal antibody, Medical & Biological Laboratories, Nagoya, Japan) and immunostaining method analysis, which may explain the discrepancies [24]. IL-33 is able to increase PD-1 and PD-L1 expression at the surface of CD8+ T lymphocytes and cancer cells, respectively [22]. The process is most likely driven via enhanced T cell production of IFN-γ. However, we did not find any association between the expression of PD-L1 and IL-33 in our cohort.

Unfortunately, our study has several limitations. The cohort size is suboptimal and the number of cases representing various compartments is low. Thus, it is impossible to draw definite conclusions regarding the survival analysis.

## 5. Conclusions

PD-L1 was commonly expressed in OSCC by TCs and TILs in our cohort. However, OSCC immunobiology and prognostic significance of PD-L1 expression vary depending on tumor location. High PD-L1 expression on TILs was strongly correlated with the lack of nodal metastases and, thus, better OS in all locations. On the other hand, PD-L1 expression on TCs seems to have a distinct impact on survival in cancers of the tongue and floor of the oral cavity and other locations. Our findings have the potential to be applied in clinical practice for better post-operative OSCC monitoring. Nevertheless, it is unknown if this factor may influence the response to immune checkpoint blockade. IL-33 expression was rarely observed and had no prognostic significance but its expression on TCs was significantly associated with tumor location. It supports site-specific variations in TME of oral cancer. Thus, future research on the immune landscape of OSCC and its responsiveness to immune therapy should focus on the analysis of cancers from distinct compartments of the oral cavity.

## Figures and Tables

**Table 1:** The summary of clinicopathological features with relation to PD-L1 expression on TCs and TILs (*p*—*p* value; *—statistically significant *p*). *p* values were calculated with chi square.

Parameters	Case Number n (%)	PD-L1 on TCs			PD-L1 on TILs		
		Low n (%)	High n (%)	*p*	Low n (%)	High n (%)	*p*
**Gender**							
Female	32 (33.68)	16 (16.84)	16 (16.84)	0.607	20 (21.05)	12 (12.63)	0.292
Male	63 (66.32)	35 (36.84)	28 (29.47)		46 (48.42)	17 (17.89)	
**Smoking**							
No	22 (30.14)	11 (15.07)	11 (15.07)	0.939	15 (20.55)	7 (10.27)	0.548
Yes	51 (69.86)	26 (35.62)	25 (34.25)		31 (42.47)	20 (27.40)	
**Alcohol**							
No	58 (80.56)	29 (40.28)	29 (40.28)	1.000	35 (48.61)	23 (31.94)	0.415
Yes	14 (19.44)	7 (9.72)	7 (9.72)		10 (13.70)	4 (5.56)	
**Grade**							
1	39 (42.05)	22 (23.16)	17 (17.89)	0.656	26 (27.37)	13 (13.68)	0.620
2–3	56 (58.95)	29 (30.53)	27 (28.42)		40 (42.11)	16 (16.84)	
**Stage**							
I–II	32 (42.67)	17 (22.08)	15 (15.79)	0.832	14 (18.18)	18 (18.18)	0.004 *
III–IV	45 (57.33)	25 (32.47)	20 (25.97)		34 (44.16)	11 (14.29)	
**T**							
1–2	42 (55.26)	23 (30.26)	18 (23.68)	0.874	23 (30.26)	19 (25.00)	0.157
3–4	34 (44.74)	18 (23.68)	16 (21.05)		24 (31.58)	10 (10.53)	
**N**							
0	39 (52.70)	17 (22.97)	22 (29.73)	0.097	18 (24.32)	21 (28.38)	0.006 *
1–3	35 (47.30)	22 (29.73)	13 (17.57)		27 (36.49)	8 (10.81)	
**Location**							
Tongue/ Floor of the oral cavity	55 (57.89)	35 (36.84)	20 (21.05)	0.022 *	37 (38.95)	18 (18.95)	0.584
Other	40 (42.11)	16 (16.84)	24 (25.26)		29 (30.53)	11 (11.58)	

**Table 2:** The univariate and multivariate Cox’s proportional hazard analysis (HR—hazard ratio; 95% CI—confidence interval; *p*—*p* value; *—statistically significant *p*).

Feature	Univariate Cox’s Proportional Hazard Analysis		Multivariate Cox Regression Model	
	HR (95% CI)	*p*	HR (95% CI)	*p*
Gender (Male vs. Female)	1.026 (0.626–1.681)	0.919		
Smoking (No vs. Yes)	0.738 (0.392–1.389)	0.347		
Alcohol (No vs. Yes)	1.001 (0.485–2.065)	0.997		
Location (Tongue/floor of the oral cavity vs. other)	0.957 (0.596–1.537)	0.855		
Grade (2–3 vs. 1)	1.767 (1.074–2.906)	0.025 *		
Stage (1–2 vs. 3–4)	0.333 (0.185–0.598)	<0.001 *		
T (1–2 vs. 3–4)	0.383 (0.221–0.664)	<0.001 *		
N (0 vs. 1–3)	0.343 (0.196–0.600)	<0.001 *	0.345 (0.193–0.617)	<0.001
PD-L1 TCs (high vs. low)	0.991 (0.618–1.589)	0.971		
PD-L1 TILs (high vs. low)	0.525 (0.306–0.902)	0.012 *		
IL-33 TCs (positive vs. negative)	0.837 (0.428–1.637)	0.603		
IL-33 TILs (positive vs. negative)	1.036 (0.566–1.894)	0.909		

**Table 3:** The multivariate Cox’s regression models (HR—hazard ratio; 95% CI—confidence interval; *p*—*p* value; *—statistically significant *p*).

Multivariate Cox Regression Model		
Feature	HR (95% CI)	*p*
**Tongue/Floor of the oral cavity**		
T (1–2 vs. 3–4)	0.229 (0.101–0.518)	<0.001 *
PD-L1 TCs (high vs. low)	2.514 (1.977–5.282)	0.014 *
**Other locations**		
N (0 vs. 1–3)	0.239 (0.081–0.699)	0.008 *

## Data Availability

The data presented in this study are available on request from the corresponding author. The data are not publicly available due to privacy restrictions.

## Supplementary Materials

Supplementary materials can be found at https://www.mdpi.com/article/10.3390/biomedicines9091132/s1, Figure S1: Overall survival probability curves according to IL-33 expression on TILs (A) and TCs (B) in the whole cohort, Figure S2: Overall survival probability curves according to IL-33 expression on TILs (A) and TCs (B) in cancers of the tongue/floor of the oral cavity, Figure S3: Overall survival probability curves according to IL-33 expression on TILs (A) and TCs (B) in cancers of other oral compartments, Table S1: The summary of clinicopathological features with relation to IL-33 expression on TCs and TILs, Table S2: The summary of studies investigating immunohistochemical expression of PD-L1 in OSCC.

## Abbreviations

AJCC	American Joint Committee on Cancer
CAFs	carcinoma-associated fibroblasts
CI	confidence interval
FFPE	formalin-fixed paraffin-embedded
HR	hazard ratio
IHC	immunohistochemistry
IL-33	interleukin 33
IFN-γ	interferon gamma
M	lymph nodes
N	metastasis
OS	overall survival
OSCC	oral squamous cell carcinoma
*p*	*p* value
PD-L1	programmed cell death-ligand 1
ST2	membrane receptor soluble interleukin 1 receptor-like 1
T	tumor
TCs	tumor cells
TILs	tumor infiltrating lymphocytes
TMA	tissue microarray
TME	tumor microenviroment
TNM	tumor, lymph nodes, metastasis—TNM Classification of Malignant Tumors
