# Supplementary material for: High PD-L1 Expression on Tumor Cells Indicates Worse Overall Survival in Advanced Oral Squamous Cell Carcinomas of the Tongue and the Floor of the Mouth but Not in Other Oral Compartments
Source: Biomedicines. 2021 Sep 1;9(9):1132. doi: 10.3390/biomedicines9091132 (PMC8471659; doi:10.3390/biomedicines9091132)
Supplement: Supplementary file 1 [file biomedicines-09-01132-s001.zip › Supplementary Figures_29.08.2021.pdf]

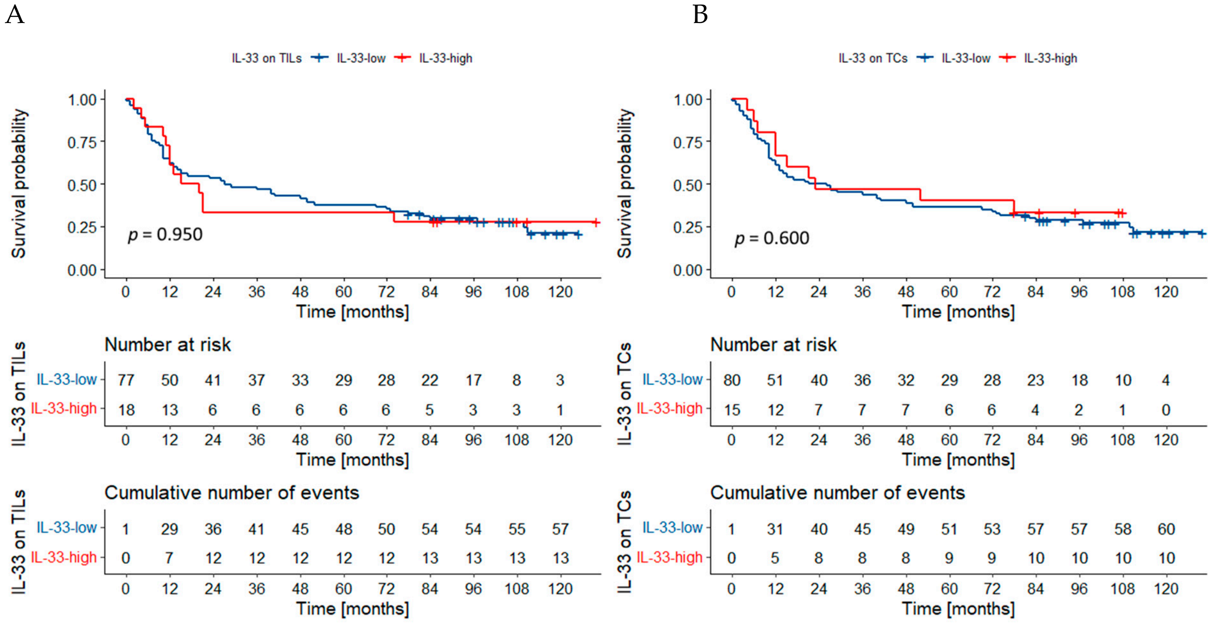

Supplementary Figure S1. Overall survival probability curves according to IL-33 expression on TILs (**A**) and TCs (**B**) in the whole cohort. Abbreviations: TCs – tumor cells; TILs – tumor infiltrating lymphocytes.

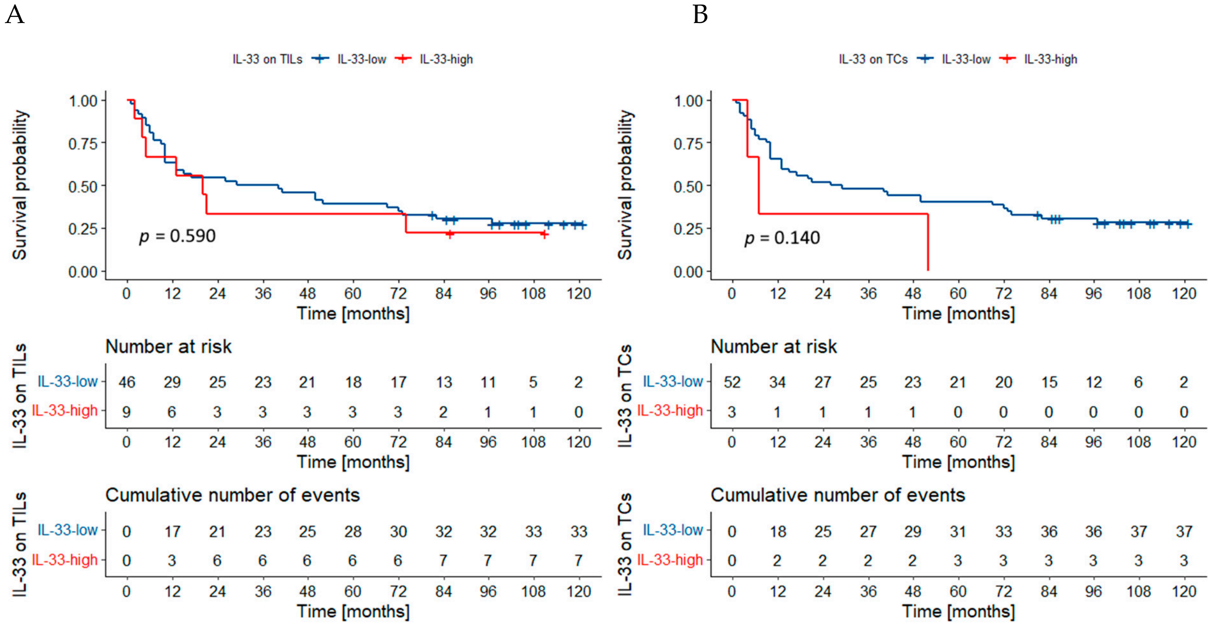

Supplementary Figure S2. Overall survival probability curves according to IL-33 expression on TILs (**A**) and TCs (**B**) in cancers of the tongue/floor of the oral cavity. Abbreviations: TCs – tumor cells; TILs – tumor infiltrating lymphocytes.

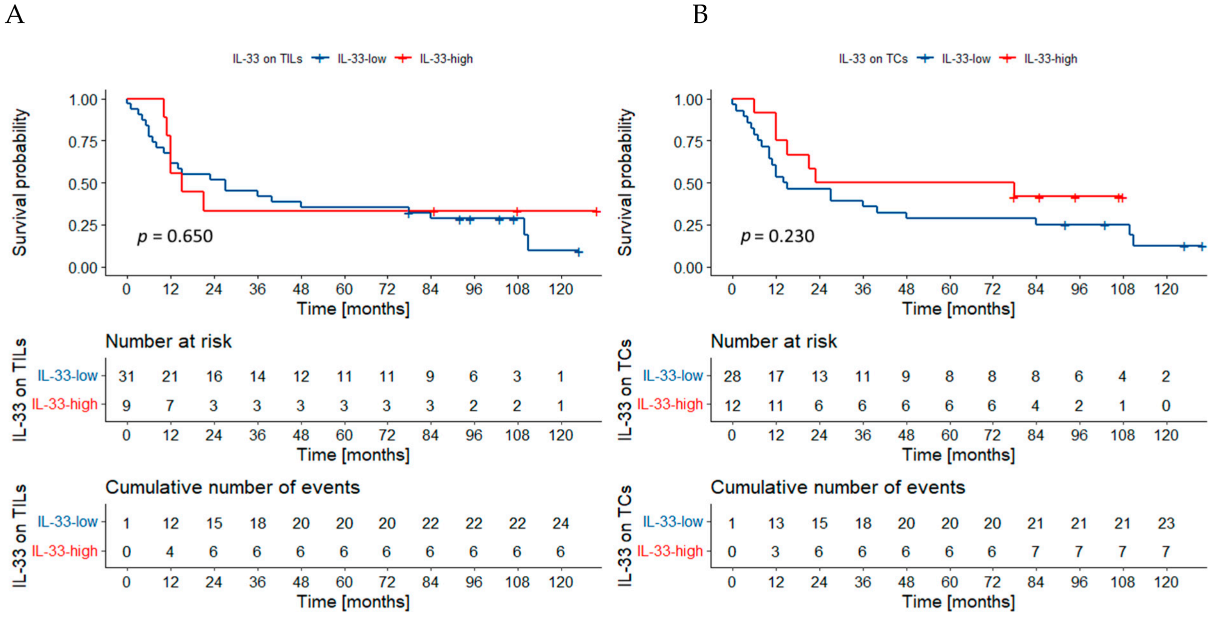

Supplementary Figure S3. Overall survival probability curves according to IL-33 expression on TILs (**A**) and TCs (**B**) in cancers of other oral compartments. Abbreviations: TCs – tumor cells; TILs – tumor infiltrating lymphocytes.
