# Supplementary material for: High PD-L1 Expression on Tumor Cells Indicates Worse Overall Survival in Advanced Oral Squamous Cell Carcinomas of the Tongue and the Floor of the Mouth but Not in Other Oral Compartments
Source: Biomedicines. 2021 Sep 1;9(9):1132. doi: 10.3390/biomedicines9091132 (PMC8471659; doi:10.3390/biomedicines9091132)
Supplement: Supplementary file 1 [file biomedicines-09-01132-s001.zip › Supplementary Tables-29.08.2021.pdf]

Supplementary Table S1. The summary of clinicopathological features with relation to IL-33 expression on TCs and TILs (*p* – *p* value; * – statistically significant *p*).

Parameters	Case number n (%)	IL-33 on TCs			IL-33 on TILs		
		Low n (%)	High n (%)	*p*	Low n (%)	High n (%)	*p*
**Gender**							
Female	32 (33.68)	29 (30.53)	3 (3.16)	0.222	28 (29.47)	4 (4.21)	0.253
Male	63 (66.32)	51 (53.68)	12 (12.63)		48 (50.52)	14 (14.74)	
**Smoking**							
No	22 (30.14)	16 (21.92)	6 (8.22)	0.165	16 (21.92)	6 (8.22)	0.467
Yes	51 (69.86)	44 (60.27)	7 (9.59)		41 (56.16)	10 (13.70)	
**Alcohol**							
No	58 (80.56)	49 (68.06)	9 (12.05)	0.594	47 (65.28)	11 (15.28)	0.427
Yes	14 (19.44)	9 (12.05)	3 (4.17)		10 (13.89)	4 (5.56)	
**Grade**							
1	39 (42.05)	35 (36.84)	4 (4.21)	0.217	34 (35.79)	5 (5.26)	0.203
2-3	56 (58.95)	45 (47.37)	11 (11.58)		43 (45.26)	13 (13.68)	
**Stage**							
I-II	32 (42.67)	30 (38.96)	2 (2.60)	0.057	27 (35.06)	5 (6.49)	0.803
III-IV	45 (57.33)	35 (45.45)	10 (12.99)		37 (48.05)	8 (10.39)	
**T**							
1-2	42 (55.26)	37 (48.68)	5 (6.58)	0.302	34 (44.74)	8 (10.53)	0.617
3-4	34 (44.74)	27 (35.53)	7 (9.21)		29 (38.16)	5 (6.58)	
**N**							
0	39 (52.70)	39 (52.70)	4 (5.41)	0.142	32 (43.24)	7 (9.46)	0.927
1-3	35 (47.30)	35 (47.30)	8 (10.81)		29 (39.19)	6 (8.11)	
**Location**							
Tongue/ Floor of the oral cavity	55 (57.89)	52 (54.74)	3 (3.16)	0.001 *	46 (48.42)	9 (9.47)	0.451
Other	40 (42.11)	28 (29.47)	12 (12.63)		31 (32.63)	9 (9.47)	

Supplementary Table S2. The summary of studies investigating immunohistochemical expression of PD-L1 in OSCC. Abbreviations: (TCs – tumor cells, TILs – tumor infiltrating lymphocytes, NE – not examined, NS – not significant, NA – not available, OS – overall survival, PP – poor prognosis, DSS – disease-specific survival, DSD – disease-specific death; DFS – disease-free survival, PFS – progression-free survival, RFS – recurrence-free survival).

No	Reference	Clone	Number of cases	IHC staining assessment methods	Assessment of PD-L1 expression in tumor cells	Number of positive cases – n (%)	Assessment of PD-L1 expression in immune cells	n (%)	Localization assessment	Other findings	Prognostic significance
1	Lequerica-Fernández et al. 2021 [33]	22C3 and E1L3N	125	Percentage of positive cells (cut-off >10%)	Yes	18 (14.4) – 22C3<br>12 (9.7) - E1L3N	No	-	Yes (NS)	High PD-L1 expression was associated with a high tumoral CD8+/FOXP3+ TILs ratio	High PD-L1 expression on TCs associated with poor DSS
2	Cui et al. 2020 [34]	E1L3N	34	H-score	Yes	NA	No	-	NE	PKD3 regulates the expression of PD-L1 triggered by IFN-γ	NE
3	Meehan et al. 2020 [35]	22C3<br>SP263	67	Percentage of positive cells (tumor proportion score)	Yes	NA	No	-	Only tongue	Recurrent tumors were characterized by higher PD-L1 expression on TCs	NE
4	Quan et al. 2020 [36]	E1L3N	159	Density of cells	No	NA	Yes	-	NE	Higher density of PD-L1-positive TILs in larger tumors	NS
5	Wilms et al. 2020 [37]	SP263	101	Quick score (ranging 0-18)	Yes	80 (79.2)	Yes	117 (96.7%)	Only tongue	Association with gender	Low PD-L1 expression on TCs associated with longer OS and DSS in females
6	Zhao et al. 2020 [38]	28-8	46	Percentage of positive cells (cut-off > 50%)	Yes	30 (65.2)	No	-	Only tongue	High PD-L1 on TCs associated with nodal metastases and higher stage	NE
7	Ahmadi et al. 2019 [39]	E1L3N	255	Percentage of positive cells (cut-off > 1%)	Yes	70 (27.5)	No	-	No	Association with gender, smoking, and p53 expression	TCs: OS – NS<br>DSS – NS<br>DFS – NS
8	de Vincente et al. 2019 [40]	E1L3N	125	Percentage of positive TCs cut-off 1%	Yes	4 (3.2)	No	-	Yes	NS	TCs: DFS – S

No	Reference	Clone	Number of cases	IHC staining assessment methods	Assessment of PD-L1 expression in tumor cells	Number of positive cases – n (%)	Assessment of PD-L1 expression in immune cells	n (%)	Localization assessment	Other findings	Prognostic significance
9	Kouketsu et al. 2019 [41]	SP142	106	Semiquantitative evaluation	Yes	73 (68.9)	No	-	No	Association with stage	TCs: NS
10	Takahashi et al. 2019 [42]	E1L3N	77	Percentage of positive cells (cut-off > 10%)	Yes	46 (60)	No	-	No	NS	TCs: OS – NS
11	Tojyo et al. 2019 [43]	SP263	49	Percentage of positive cells (cut-off ≥ 5% of TCs and ≥1% of TILs)	Yes	24 (48.9)	Yes	28 (57.1%)	No	PD-L1 expression on TCs correlated with p53 expression	TCs, TILs: DSS – NS
12	Hanna et al. 2018 [44]	9A11	81 (23 females)	Percentage of positive cells (cut-off ≥ 10%)	Yes	20/23 females (86.9)	Yes	0 (0%)	No	NS	TCs: low PD-L1 associated with worse OS in females
13	Maruse et al. 2018 [45]	E1L3N	97	Percentage of positive cells (cut-off ≥ 5%)	Yes	63 (64.9)	No	-	NE	PD-L1 expression associated with nodal and distant metastases	TCs: high PD-L1 expression associated with worse DSS
14	Stasikowska-Kanicka et al. 2018 (1) [46]	28-8	78	Semiquantitative evaluation	Yes	62 (79)	Detected	NA	NE	-	TCs: higher PD-L1 expression in poor prognosis group
15	Stasikowska-Kanicka (2) et al. 2018 [47]	28-8	70	Semiquantitative evaluation	Yes	67 (96)	Yes	13	NE	NE	NE
16	Udeabor et al. 2018 [48]	28-8	20	Semiquantitative evaluation	Yes	NA	Yes	No data	NE	NE	NE
17	Wirsing et al. 2018 [49]	SP263	45	Percentage of positive cells (cut-off ≥ 10%)	Yes	18 (40)	Detected	NA	NE	PD-L1 expression association with tumor size and density of CD4+ cells	TCs: DSS – NS
18	Ahn et al. 2017 [50]	ab153991 (polyclonal)	68	Semiquantitative evaluation	Yes	45 (66.2)	No	-	NE	Prognostic significance of PD-L1 was dependent on the	TCs: high PD-L1 ex-

No	Reference	Clone	Number of cases	IHC staining assessment methods	Assessment of PD-L1 expression in tumor cells	Number of positive cases – n (%)	Assessment of PD-L1 expression in immune cells	n (%)	Localization assessment	Other findings	Prognostic significance
										levels of miR-197	pression associated with better OS
19	Feng et al. 2017 [51]	E1L3N	119	Semiquantitative evaluation	Yes	NA	Yes	NA	NE	-	A high number of PD-L1-positive cells in the vicinity of CD8+ T-cells indicates worse OS
20	Foy et al. 2017 [52]	SP142 and 28.8	44	Percentage of positive cells (various cut-offs ≥ 1, ≥ 5, and ≥ 10%)	Yes	Dependent on antibody and threshold	Detected	NA	NE	Higher PD-L1 expression in TCs in never-smokers and never-drinkers	NE
21	Groeger et al. 2017 [53]	MIH1	15	No data	Yes	15 (100)	No	-	NE	NE	NE
22	Hirai et al. 2017 [54]	NA	24	Semiquantitative evaluation	Yes	NA	Detected	NA	NE	Association with mode of invasion	NE
23	Kogashiwa et al. 2017 [55]	SP142	84	Percentage of positive cells (cut-off ≥ 5%)	Yes	44 (52.4)	Yes	NA	NE	Unclear if expression was evaluated in TCs only or combined in TCs and TILs	High PD-L1 expression associated with better PFS and OS
24	Kubota et al. 2017 [56]	E1L3N	46	NA	No	NA	Yes	-	NE	CD163+CD204+ macrophages promote T-cell apoptosis and immunosuppression *via* IL-10 and PD-L1	NE
25	Mattox et al. 2017 [57]	5H1	53	Percentage of positive TCs and/or TILs (cut-off 1%)	Yes	42 (79.2)	Yes	35 (83.3%)	Only tongue	CD4+PD-1+ and CD8+PD-1+ TILs were functionally	OS – NS

No	Reference	Clone	Number of cases	IHC staining assessment methods	Assessment of PD-L1 expression in tumor cells	Number of positive cases – n (%)	Assessment of PD-L1 expression in immune cells	n (%)	Localization assessment	Other findings	Prognostic significance
										anergic in the presence of PD-L1+ macrophages	
26	Takakura et al. 2017 [58]	27A2	10 chemo-naïve tumors + 8 tumors treated with chemotherapy	Percentage of positive cells (cut-off 25%)	Yes	8 (80)<br>2 (25)	No	-	NE	OSCCs treated with neoadjuvant chemotherapy have lower PD-L1 expression than chemonaïve tumors	NE
27	Troeltzsch et al. 2017 [59]	E1L3N	88	Percentage of positive cells (cut-off 5%)	Yes	26 (29.5)	No	-	Yes, PD-L1 expression was more common in mandibular localisation and tongue than in maxilla and palate	High PD-L1 expression associated with nodal metastases	TCs: DSD – NS
28	Satgunaseelan et al. 2016 [60]	E1L3N	217	Percentage of positive cells (cut-off 5%) and semiquantitative evaluation	Yes	40 (18.4)	No	-	Yes, PD-L1 expression was more frequent in the buccal mucosa and the tongue compared to the gingiva and the floor of mouth	Association with gender	TCs: DSS – NS<br>DFS – NS<br>OS – NS

No	Reference	Clone	Number of cases	IHC staining assessment methods	Assessment of PD-L1 expression in tumor cells	Number of positive cases – n (%)	Assessment of PD-L1 expression in immune cells	n (%)	Localization assessment	Other findings	Prognostic significance
29	Straub et al. 2016 [61]	E1L3N	80	Percentage of positive cells (cut-off 5%).	Yes	36 (45)	No	-	NE	NS with clinicapathological findings	TCs: High PD-L1 expression associated with worse OS and RFS
30	Chen et al. 2015 [62]	2B11D11	218	Percentage of positive cells (cut-off 5%).	Yes	139 (63.8)	No	-	NE	PD-L1 expression assessed also in metastatic lymph nodes	TCs: high PD-L1 expression and tumor necrosis associated with worse DFS and OS
31	Lin et al. 2015 [63]	GTX104763 (polyclonal)	305	Intensity of staining	Yes	134 (43.9)	No	-	NE	Association with gender	TCs: High PD-L1 expression associated with worse OS in male smokers
32	Oliveira-Costa et al. 2015 [64]	ab28753 (polyclonal)	97	Percentage of positive cells (cut-off 5%)	Yes	47 (48.4)	No	-	Yes, NS	Circulating tumor cells from selected patients express PD-L1	TCs: High cytoplasmatic expression of PD-L1 associated with better OS
33	Cho et al. 2011 [65]	ab82059	45	Semiquantitative evaluation	Yes	39 (86.7)	No	-	NE	Association with grade and density of CD8+ TILs	TCs: OS – NS
34	Malaspina et al. 2011 [66]	MIH1	39	No data	Yes	NA	No	-	NE	NE	NE
